# Bibliometric Insights of Global Research Landscape in Mitophagy

**DOI:** 10.3389/fmolb.2022.851966

**Published:** 2022-07-18

**Authors:** Guoli Li, Wei Yin, Yiya Yang, Hongyu Yang, Yinyin Chen, Yumei Liang, Weiru Zhang, Tingting Xie

**Affiliations:** ^1^ Department of Nephrology, Hunan Provincial People’s Hospital, The First Affiliated Hospital of Hunan Normal University, Changsha, China; ^2^ Changsha Clinical Research Center for Kidney Disease, Changsha, China; ^3^ Hunan Clinical Research Center for Chronic Kidney Disease, Changsha, China; ^4^ Department of Rheumatology and Immunology, Xiangya Hospital, Central South University, Changsha, China; ^5^ Department of General Medicine, Xiangya Hospital, Central South University, Changsha, China; ^6^ International Collaborative Research Center for Medical Metabolomics, Xiangya Hospital Central South University, Changsha, China; ^7^ National Clinical Research Center for Geriatric Disorders (Xiangya Hospital), Changsha, China

**Keywords:** mitophagy, bibliometric analysis, CiteSpace, VOSviewer, visualization

## Abstract

**Background:** Autophagy is a highly regulated and evolutionarily conserved process in eukaryotes which is responsible for protein and organelle degradation. Although this process was described over 60 years ago, the selective autophagy of mitochondria (mitophagy) was recently coined in 2005. Research on the topic of mitophagy has made rapid progress in the past decade, which proposed to play critical roles in human health and disease. This study aimed to visualize the scientific outputs and research trends of mitophagy.

**Methods:** Articles and reviews related to the topic of mitophagy were retrieved from the Web of Science Core Collection on 30 November 2021. Two kinds of software (CiteSpace and VOSviewer) were used to perform a visualized analysis of countries/regions, institutions, authors, journals, references, and keywords.

**Results:** From 2005 to 2021, total 5844 publications on mitophagy were identified for final analysis. The annual number of publications grew yearly over the past 17 years. United States (N = 2025) and Chinese Academy of Sciences is the leading country and institute (N = 112) ranked by the number of publications, respectively. The most productive author was Jun Ren (N = 38) and Derek P. Narendra obtained the most co-cited times (2693 times). The journals with the highest output and the highest co-citation frequency were *Autophagy* (N = 208) and *Journal of Biological Chemistry* (co-citation: 17226), respectively. Analyses of references and keywords suggested that “mechanism of mitochondrial quality control”, “molecule and signaling pathway in mitophagy”, and “mitophagy related diseases” were research hotspots, and parkin-mediated mitophagy and its roles in skeletal muscle and inflammation-related diseases may be the frontiers of future research.

**Conclusion:** Although mitophagy research has flourished and attracted attention from all over the world, the regional imbalance in the development of mitophagy research was observed. Our results provided a comprehensive global research landscape of mitophagy from 2005– 2021 from a perspective of bibliometrics, which may serve as a reference for future mitophagy studies.

## Introduction

As the powerhouse of the cell, mitochondria play essential roles in regulating cellular metabolism and physiology (Harper et al., 2018). Thus, the mitochondrial quality control is important to maintain the life of cells, as well as human health (Pickles et al., 2018). Mitophagy was a recently identified form of selective autophagy, during which dysfunctional or superfluous mitochondria were removed (Gustafsson and Dorn, 2019a). Although mitophagy was described firstly in yeast, it is an evolutionarily conserved mechanism that has also been identified in mammals (Priault et al., 2005; Palikaras et al., 2018). So far, at least two distinct pathways have been found to be involved in the mechanisms of mitophagy. One is the PINK1/Parkin pathway, and the other is the mitophagy receptor pathway (Gustafsson et al., 2019b); its functional roles include mitochondrial quality and quantity control, metabolic reprogramming, and differentiation (Palikaras et al., 2018). For more than a decade, significant progress has been made in the field of mitophagy and a list of diseases, such as neurodegenerative disease (Malpartida et al., 2021), cardiovascular disease (Bravo-San Pedro et al., 2017), and cancer (Panigrahi et al., 2020), were found to be closely associated with mitophagy impairment. Recently, several systematic reviews on mitophagy have been performed (Palikaras et al., 2018; Pickles et al., 2018; Onishi et al., 2021). With these reviews, we can understand the mechanism, function, and related diseases of mitophagy. However, the comprehensive knowledge of the global research status, current hotspots, and future trends of mitophagy is still lacking.

Bibliometric analysis is a powerful approach that uses literature metrics or indicators to quantitatively measure the research performance in a certain field (Deng et al., 2020; Wang et al., 2021c). It was coined more than 50 years ago (Pritchard, 1969), and now it has been widely used as a methodology for evaluating trends and frontiers from a large number of publications (Zhao et al., 2020; Hu et al., 2021). Through bibliometric analysis, influential articles, main research fields, and new research directions can be timely and comprehensively achieved by researchers (Thompson and Walker, 2015). Compared with traditional reviews, bibliometric-based analyses can provide a more comprehensive perspective on the research trends, and the data is more objective (Yan et al., 2021). With the development of bibliometrics, dozens of software tools for conducting bibliometric analysis are available for researchers (Moral-Muñoz et al., 2020). CiteSpace and VOSviewer represent the two most commonly used tools for visualization (Moral-Muñoz et al., 2020). Recently, thousands of bibliometric studies have been conducted in a range of fields (Donthu et al., 2021), while only one study reported on mitophagy so far which focused on comparing the difference in research progress in China and other developed countries (Chen et al., 2021). The global research status of mitophagy remains largely unknown.

In this study, a bibliometric-based analysis was performed to systematically evaluate the mitophagy studies from 2005 to 2021. By taking advantage of CiteSpace and VOSviewer, we comprehensively analyzed the publication output, disciplinary composition, countries/regions, institutions, publishers, funders, authors, journals, top-cited articles, references, and appeared keywords. Our results draw a visualization map of the global research landscape of mitophagy, helping researchers, especially for those who are new to this field, to deepen their understanding of the current status and future directions of mitophagy research.

## Methods

### Data Collection and Cleaning

**FIGURE 1:**
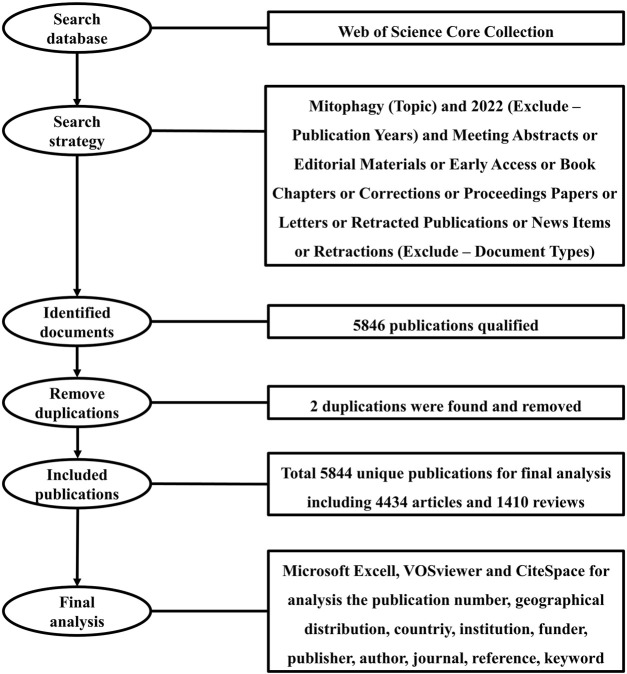
Flowchart of the methodology and design of this study.

Publications were extracted from the database of the Web of Science Core Collection (WoSCC) in this study. WoSCC is the world’s oldest, most widely used, and authoritative database of research publications and citations, which contains records of articles from the highest impact journals worldwide (Birkle et al., 2020). A literature search was performed within 1 day on 30 November 2021. The search formula was set as follows: Mitophagy (Topic) and 2022 (Exclude–Publication Years) and Meeting Abstracts or Editorial Materials or Early Access or Book Chapters or Corrections or Proceedings Papers or Letters or Retracted Publications or News Items or Retractions (Exclude–Document Types). A search by Topic will be searched in the title, abstract, author keywords, and keywords plus. A total of 5846 literatures were obtained and exported for full record and cited references in the format of plain text files. Using Endnote software, a total of two duplications were found. After removing duplications, a total of 5844 unique records remained, including 4434 articles and 1410 reviews (Figure 1). Research articles published original findings and reviews and are written based on existing articles. Based on these two types of documents, the impact factor (IF) of an academic journal is calculated by Clarivate. Therefore, consistent with other studies (Chen et al., 2020; Hu et al., 2021; Zhang et al., 2021), we only used articles and reviews for bibliometric analysis in this study.

Of note, we have cleaned the data before analysis (Wang et al., 2021b; Zhang et al., 2021). These included: 1) Publications from Taiwan and the People’s Republic of China were reclassified to China; 2) Publications from England, Scotland, Northern Ireland, and Wales were assigned to the United Kingdom; 3) Publications from Austria, Belgium, Bulgaria, Croatia, Cyprus, Czech Republic, Denmark. Estonia, Finland, France, Germany, Greece, Hungary, Ireland, Italy, Latvia, Lithuania, Luxembourg, Malta, Netherlands, Poland, Portugal, Romania, Slovakia, Slovenia, Spain, and Sweden were merged into European Union; 4) Publications from Cent S Univ and Cent South Univ merged into Cent South Univ; 5) Publications from Guangdong Med Coll and Guangdong Med Univ merged into Guangdong Med Univ; 6) Publications from Univ Texas Southwestern Med Ctr Dallas and Univ Texas SW Med Ctr Dallas merged into Univ Texas Southwestern Med Ctr Dallas.

### Visualized Analysis

CiteSpace software (Drexel University, Philadelphia, PA, United States) is a freely available Java application, which was widely used for visualizing and analyzing trends and patterns in the scientific literature (Chen, 2006). It was designed by Chen (2004) and last updated on 17 January 2021 (Version 5.8. R2). Web of Science is the primary source of input data for CiteSpace. It has been widely used for visualizing bibliometric networks by other studies (Hu et al., 2021; Ma et al., 2021). In this study, CiteSpace was used to perform a bibliometric analysis of collaborations (countries/regions, institutions, authors), the timeline view of co-occurrence (keywords), and citation bursts (references and keywords). Co-occurrence analysis was used to find the number of terms where they occur together in the same article and weighted by the frequency of occurrence. Citation bursts means that the frequency of a term has soared within a certain short period. The primary parameters were set as follows: time slicing (2005–2021), years per slice (2 years), and selection criteria (g-index, k = 25). Other parameters were set according to the CiteSpace manual for different situations.

VOSviewer software is a useful tool for constructing and visualizing bibliometric networks (Van Eck and Waltman, 2010). It was developed by the Center for Science and Technology Research at Leiden University (The Netherlands) in 2007 (Van Eck and Waltman, 2007). The latest version 1.6.17 was released on 22 July 2021, and is free for download. Co-authorship networks, citation-based networks, and co-occurrence networks can be created based on data downloaded from the Web of Science. It also has been widely used for visualizing bibliometric networks by other groups (Chen et al., 2020; Xiong et al., 2021). In this study, VOSviewer was used for visualizing the co-authorship between countries/regions, institutions, authors, and the co-occurrence of keywords.

Other information such as impact factor (IF) and Journal Citation Reports (JCR) division of journals were obtained from the Web of Science website (www.webofscience.com) directly on 30 November 2021. Microsoft Office Excel 2016 was used to analyze the annual publications. WordArt (https://wordart.com/) was used to create the word cloud of the subject categories. GunnMap 2 (http://gunnmap.herokuapp.com/) was used to display the global publication distribution on a world map.

## Results

### Analysis of Publication Trends and Subject Categories

**FIGURE 2:**
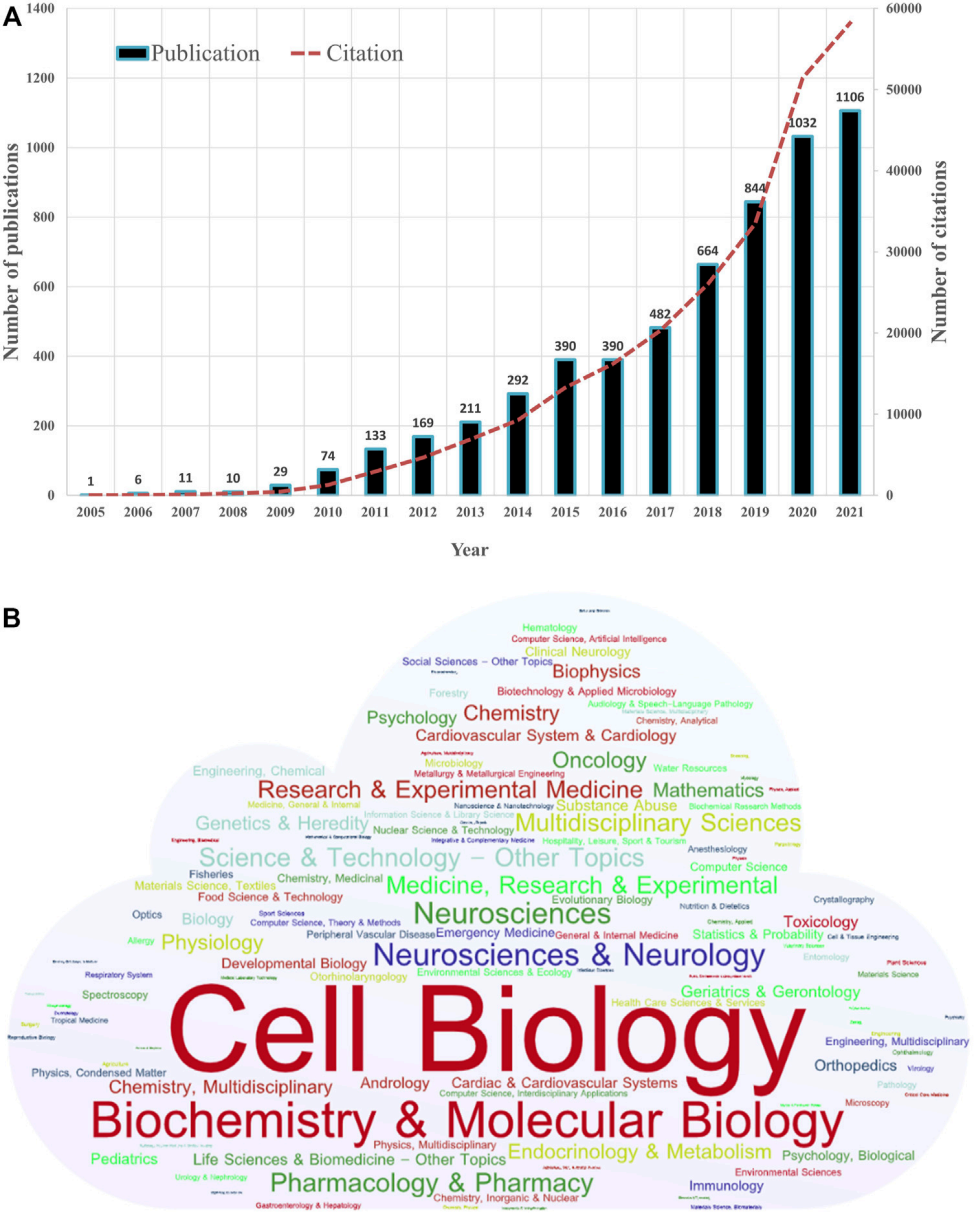
Distribution of publications on mitophagy by year and category. **(A)** Number of annual publications and citations of mitophagy from 2005 to 2021 globally. **(B)** The word cloud map of the subject categories on mitophagy (Created with https://wordart.com/).

From the first article published on 10 June 2005 to 30 November 2021, a total of 5844 publications on mitophagy research were identified. Figure 2A shows the annual number of publications and citations worldwide. In general, the annual number of publications increased year by year. The early stage (2005–2014) saw a relatively stable growth, with a publication count of no more than 300 per year. While a steep growth was observed since 2015. Up to date (30 November 2021), the publication outputs have reached over 1100 in 2021. The number of 2020 (1032) was over 27 times that of 2010 (74). The annual number of citations were also increased yearly, and the number in 2020 (51433) was over 40 times that of 2010 (1281).

By the analysis of CiteSpace, we identified 127 disciplinary categories involved in the field of mitophagy (Figure 2B). Through WordArt, a map of the word cloud of the subject categories was created (the more publications belong to the category, the bigger the word appears in the word cloud). Cell Biology (1916, 32.79%) is the most common category, followed by Biochemistry and Molecular Biology (1574, 26.93%), Neurosciences and Neurology (616, 10.54%), Neurosciences (566, 9.69%) and Pharmacology and Pharmacy (437, 7.48%).

### Analysis of Countries and Institutions

**FIGURE 3:**
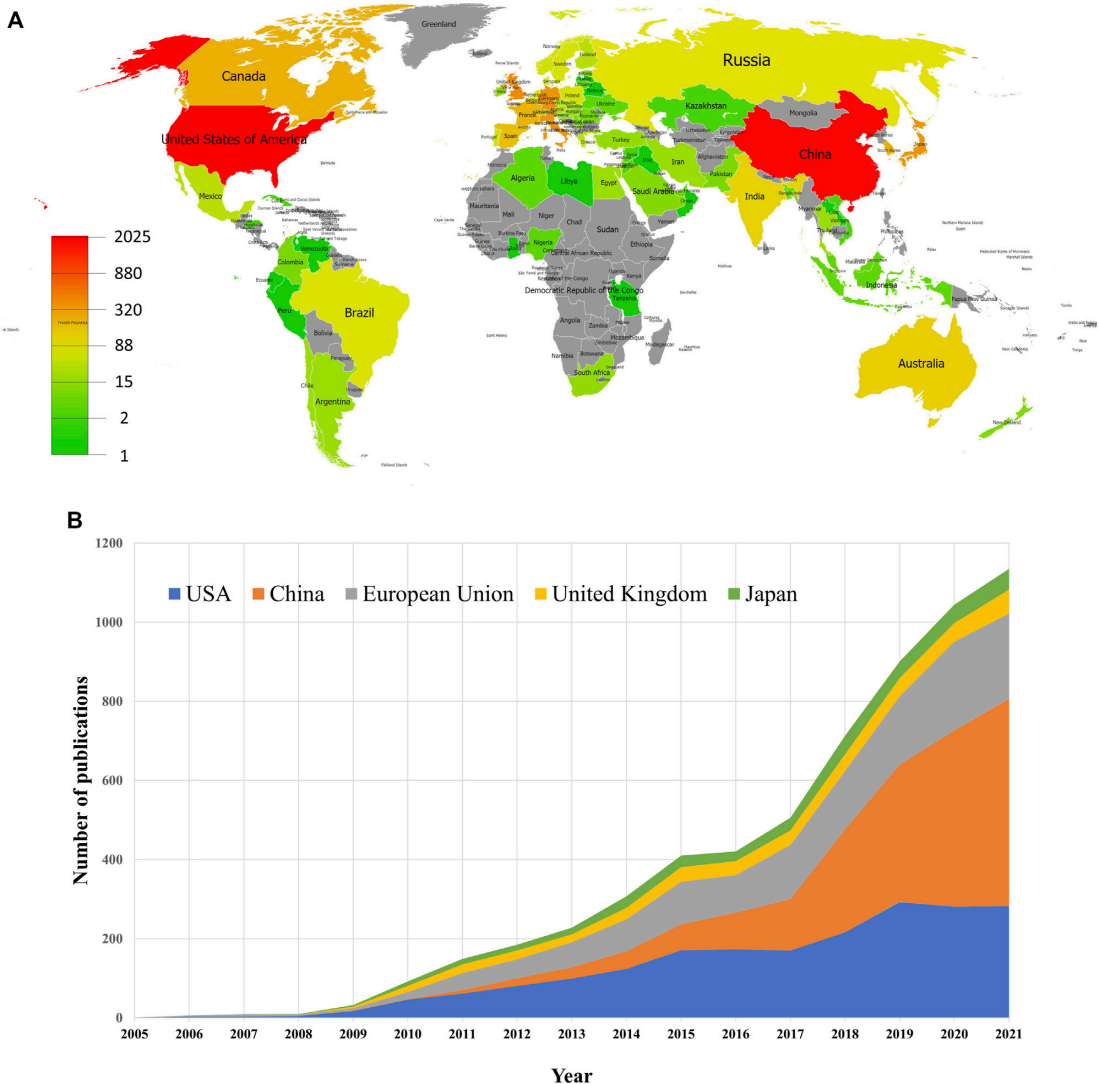
Geographical distribution of publications on mitophagy. **(A)** Geographical distribution map of publications on mitophagy. **(B)** Trends of the annual publications of mitophagy in the United States, China, European Union, United Kingdom, and Japan.

Figure 3A shows the geographical distribution of global productivity of studies on mitophagy. A total of 76 different countries/regions covering five continents have taken part in mitophagy research. Thirteen countries/regions published only one paper, and 12 countries/regions published over 100 articles. These results indicated that mitophagy research has attracted attention from all over the world. However, research development of mitophagy in different countries/regions is uneven. Among the top 10 most prolific countries or political entities (Table 1), United States had the highest number of publications (2025, 34.65%), followed by China (1968, 33.68%), the European Union (1373, 23.49%), the United Kingdom (418, 7.15%), and Japan (364, 6.23%). These top five countries or political entities account for most of the total publications. From Figure 3B, we can see that the number of annual publications slowly increased after 2015 in the United States, the European Union, the United Kingdom, and Japan. Whereas, a relatively sharp rise was witnessed in China after 2015, especially during the year from 2017 to 2021. Therefore, we can conclude that China has been the main driver of the increase in mitophagy research since 2015. Among the top 10 most prolific institutions, six of them come from China, and the remaining institutions come from the United States (N = 2), the United Kingdom (N = 1), and Canada (N = 1). Chinese Acad Sci (112, 1.92%) is the leading institute, followed by Univ Pittsburgh (109, 1.87%), Univ Calif San Diego (92, 1.57%) and Zhejiang Univ (88, 1.51%). Of note, no institutions from the European Union appeared on the list.

**TABLE 1:** Top 10 countries/political entities and institutions contributed to publications on mitophagy.

Rank	Country	Year	Centrality	Count (%)	Rank	Institution (Country)	Year	Centrality	Count (%)
1	United States	2006	0.51	2025 (34.65%)	1	Chinese Acad Sci (China)	2010	0.03	112 (1.92%)
2	China	2009	0.21	1968 (33.68%)	2	Univ Pittsburgh (United States)	2007	0.13	109 (1.87%)
3	European Union	2005	0.39	1373 (23.49%)	3	Univ Calif San Diego (United States)	2009	0.05	92 (1.57%)
4	UK	2009	0.35	418 (7.15%)	4	Zhejiang Univ (China)	2013	0.04	88 (1.51%)
5	Japan	2008	0.05	364 (6.23%)	5	Fudan Univ (China)	2010	0.01	87 (1.49%)
6	South Korea	2007	0.05	242 (4.14%)	6	Cent South Univ (China)	2015	0.04	85 (1.45%)
7	Canada	2006	0.08	241 (4.12%)	7	UCL (UK)	2010	0.07	74 (1.27%)
8	Australia	2007	0.07	139 (2.38%)	8	Shanghai Jiao Tong Univ (China)	2013	0.02	73 (1.25%)
9	India	2013	0.1	129 (2.21%)	9	Sun Yat-sen Univ (China)	2013	0.03	68 (1.16%)
10	Russia	2009	0.06	98 (1.68%)	10	McGill Univ (Canada)	2011	0.03	67 (1.15%)

UK: United Kingdom. UCL: Univ College London. Year: The year in which the earliest article was published.

**FIGURE 4:**
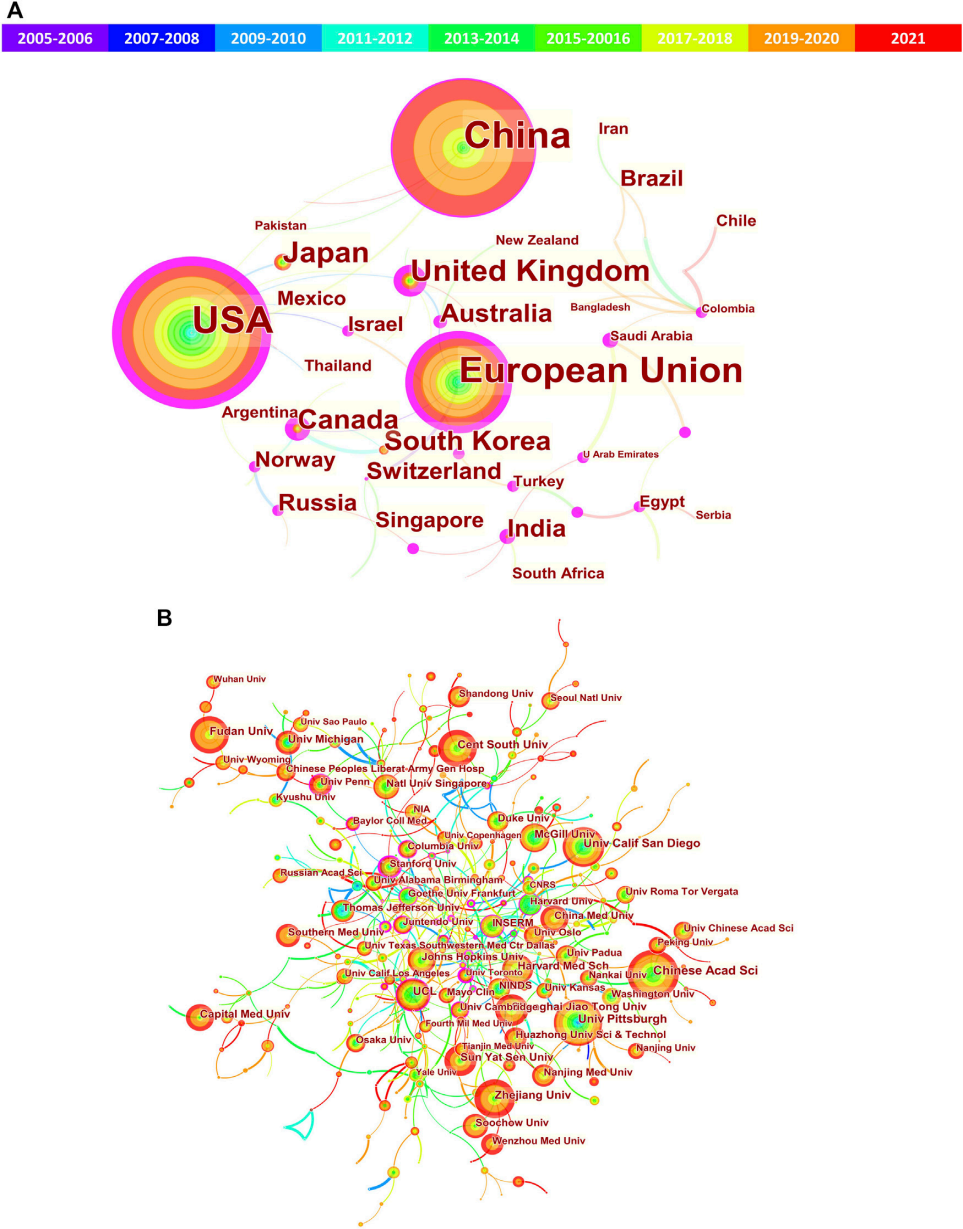
Cooperation network of the countries/regions and institutions related to mitophagy. **(A)** Network map of countries/regions; **(B)** Network map of institutions. The nodes represent countries/regions or institutions (the larger circle, the higher the number of publications); lines between the nodes represent collaboration (the wider lines, the more frequency of collaborations); the purple ring represents active cooperation (centrality≥0.10).

Next, network visualization of the co-authorship countries and institutions was analyzed *via* CiteSpace. In the cooperative network map (Figure 4), the nodes represent countries/institutions (the larger circle, the higher the number of publications); lines between the nodes represent a collaboration between two countries/institutions on the same article (the wider lines, the more frequency of collaborations); the purple ring represents active cooperation of the country/institutions (centrality≥0.10). Herein, high centrality indicates active cooperation. From Figure 4 and Table 1, we can see that United States (0.51) and Univ Pittsburgh (0.13) obtained the highest centrality among countries and institutions, respectively. In addition, many other countries or political entities, such as European Union, United Kingdom, Canada, India, Australia, Russia and Norway, also had a high centrality (purple ring). However, China and Japan do not have a high centrality, indicating their insufficient international cooperation.

### Analysis of Authors and Co-cited Authors

**FIGURE 5:**
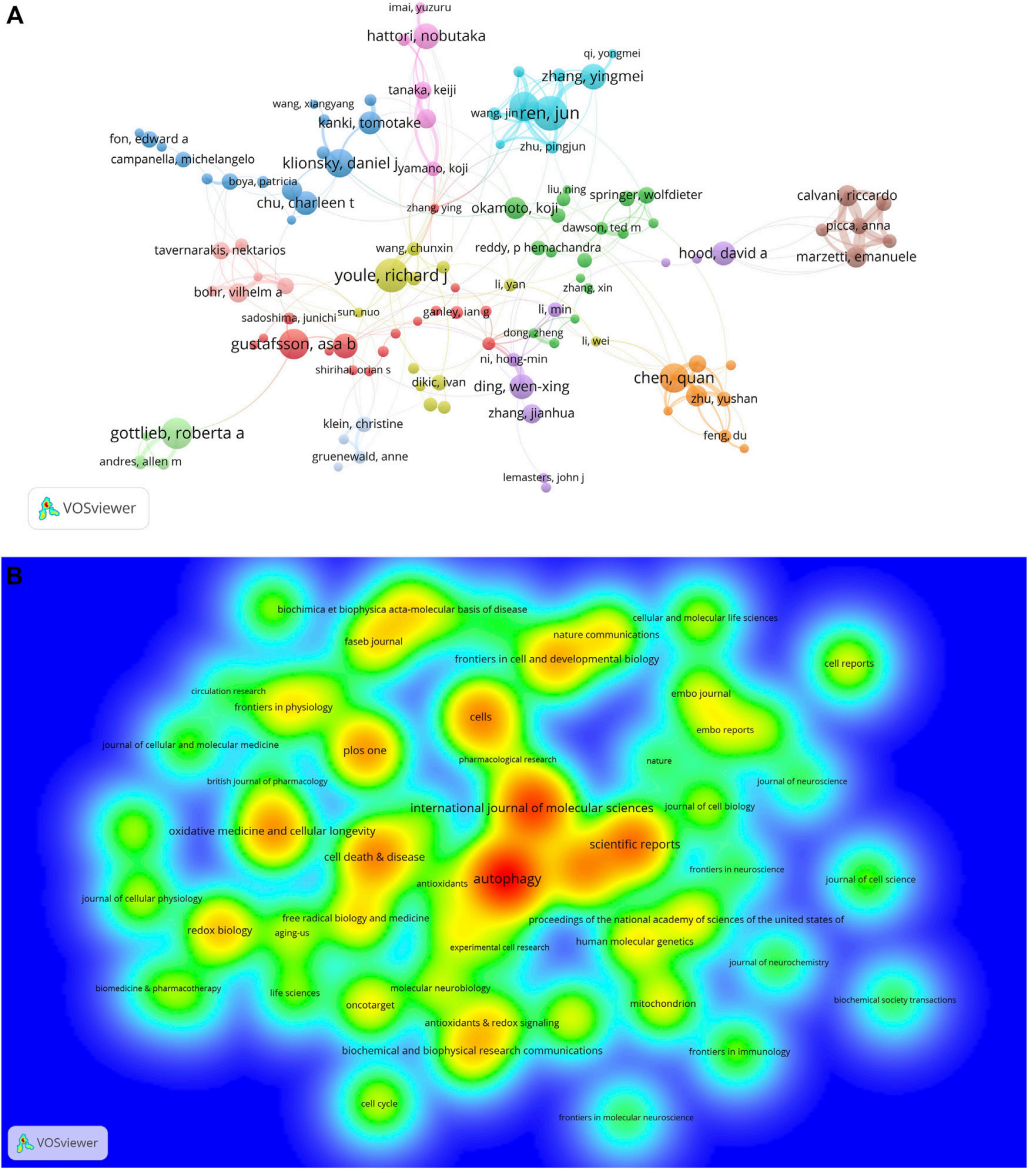
Co-authorship of authors and productive journals on mitophagy. **(A)** Co-authorship network of authors in the field of mitophagy. **(B)** Density map of top 56 most productive journals published papers on mitophagy.

A total of 27872 authors published literature on mitophagy (data not shown). Among the top 10 prolific authors (Table 2), Jun Ren published the largest number of papers (N = 38), followed by Richard J Youle (N = 37), Roberta A Gottlieb (N = 34), and Asa B Gustafsson (N = 33). In the cooperative network map (Figure 5A), the 114 authors who published at least 10 papers and had co-authorship with others were visualized. A total of 114 terms, 12 clusters, 280 links, and a total link strength of 1106 were generated. Each node represented an author. The size of the nodes is determined by the number of publications (The higher the number, the larger the node). The same color represented the same cluster. The line between the nodes represented the co-authorship between authors (The stronger the cooperation relationship, the wider the line). The number of total link strengths reflected the total co-authorship strength between authors.

**TABLE 2:** Top 10 authors and co-cited authors contributed to mitophagy research.

Rank	Author	Documents	Institutions (Countries)	Rank	Co-cited author	Co-citation	Institutions (Countries)	Countries
1	Jun Ren	38	Fudan Univ (China)	1	Derek P. Narendra	2693	NINDS (United States)	United States
2	Richard J. Youle	37	NINDS (United States)	2	Noboru Mizushima	1446	Univ Tokyo (Japan)	Univ Tokyo
3	Roberta A. Gottlieb	34	Cedars Sinai Med Ctr (United States)	3	Richard J. Youle	1313	NINDS (United States)	United States
4	Asa B. Gustafsson	33	Univ Calif San Diego (United States)	4	Hao Zhou	1295	Chinese Peoples Liberat Army Hosp (China)	China
5	Quan Chen	32	Nankai Univ (China)	5	Sven Geisler	1141	Univ Tubingen (Germany)	Germany
6	Hao Zhou	32	Chinese Peoples Liberat Army Hosp (China)	6	Michael Lazarou	1085	Monash Univ (Australia)	Australia
7	Daniel J. Klionsky	31	Univ Michigan (United States)	7	Gilad Twig	1069	Boston Univ (United States)	United States
8	Michael P. Lisanti	30	Thomas Jefferson Univ (United States)	8	Daniel J. Klionsky	940	Univ Michigan (United States)	United States
9	Federica Sotgia	29	Univ Manchester (England)	9	Hsiuchen Chen	857	Caltech (United States)	United States
10	Nobutaka Hattori	28	Juntendo Univ (Japan)	10	Noriyuki Matsuda	800	Tokyo Metropolitan Inst Med Sci (Japan)	Japan

NINDS, NIH, national institute of neurological disorders and stroke; Caltech, California Institute of Technology.

Co-citation is defined as the frequency with which two documents are cited together by another or more articles at the same time, providing a way to study the specialty structure of science (Wang et al., 2021b). In this study, a total of 106127 co-cited authors were identified (data not shown). Among the top 10 most frequently co-cited authors (Table 2), Derek P Narendra (2693 times) ranked first, followed by Noboru Mizushima (1446 times) and Richard J Youle (1313 times). It is worth noting that Richard J Youle, Hao Zhou, and Daniel J Klionsky were the top 10 authors ranked by both numbers of publication and co-citation frequency.

### Analysis of Journals and Co-Cited Journals

A total of 1021 academic journals had published articles on mitophagy (data not shown). The top 10 journals published with the highest number of publications were shown in Table 3. Among them, *Autophagy* (N = 208) most productive one and had the highest IF 2020 (16.016). Eight (80%) of the journals had an IF 2020 over 5 and 6 (60%) were located in JCR (2020) Q1 region. A density map of the top 56 productive journals (with over 20 publications) was shown in Figure 5B. The top 10 journals ranked by co-cited frequency were also shown in Table 3. We can see that the journal with the highest co-citations was *Journal of Biological Chemistry* (co-citation: 17226). Notably, *Autophagy*, *Journal of Biological Chemistry*, and *PLoS One* were the top 10 journals ranked both by a number of publications and co-cited frequency.

**TABLE 3:** Top 10 journals and co-cited journals contributed to mitophagy research.

Rank	Journal	Count	IF (2020)	JCR Category quartile	Co-cited journal	Citation	IF (2020)	JCR Category quartile
1	Autophagy	208	16.016	Cell Biology (Q1)	Journal of Biological Chemistry	17226	5.157	Biochemistry and Molecular Biology (Q2)
2	International Journal of Molecular Sciences	155	5.924	Biochemistry and Molecular Biology (Q1); Chemistry, Multidisciplinary (Q2)	PNAS*	13970	11.205	Multidisciplinary Sciences (Q1)
3	Scientific Reports	115	4.38	Multidisciplinary Sciences (Q1)	Nature	13427	49.962	Multidisciplinary Sciences (Q1)
4	Cells	103	6.6	Cell Biology (Q2)	Autophagy	12774	16.016	Cell Biology (Q1)
5	Cell Death and Disease	101	8.469	Cell Biology (Q1)	Journal of Cell Biology	10499	10.539	Cell Biology (Q1)
6	Journal of Biological Chemistry	97	5.157	Biochemistry and Molecular Biology (Q2)	Cell	9829	41.584	Biochemistry and Molecular Biology (Q1); Cell Biology (Q1)
7	Oxidative Medicine and Cellular Longevity	93	6.543	Cell Biology (Q2)	Science	8462	47.728	Multidisciplinary Sciences (Q1)
8	PLoS One	90	3.24	Multidisciplinary Sciences (Q2)	PLoS One	6957	3.24	Multidisciplinary Sciences (Q2)
9	Frontiers In Cell and Developmental Biology	78	6.684	Cell Biology (Q2); Developmental Biology (Q1)	Human Molecular Genetics	6528	6.15	Biochemistry and Molecular Biology (Q1); Genetics and Heredity (Q1)
10	Redox Biology	69	11.799	Biochemistry and Molecular Biology (Q1)	EMBO Journal	6285	11.598	Biochemistry and Molecular Biology (Q1); Cell Biology (Q1)

What are the publication patterns for mitophagy studies in general journals? In this study, we found that most articles on mitophagy were published in a cell or molecular biology journals (Table 3). Unexpectedly, the *Journal of Biological Chemistry* was the most frequently co-cited journal (co-citation: 17226) with the IF 2020 as 5.157 that far exceeded some high-impact journals, such as *Nature* (co-citation: 13427; IF 2020: 49.962), *Cell* (co-citation: 9829; IF 2020: 41.584), and *Science* (co-citation: 8462; IF 2020: 47.728). Also notably, *Autophagy* (IF 2020: 16.016), *Journal of Biological Chemistry* (IF 2020: 5.157), and *PLoS One* (IF 2020: 3.24) were the top 10 both productive and cited journals. These results provided some guidance to future authors deciding where to publish their work on mitophagy research.

### Analysis of Publishers and Funders

**FIGURE 6:**
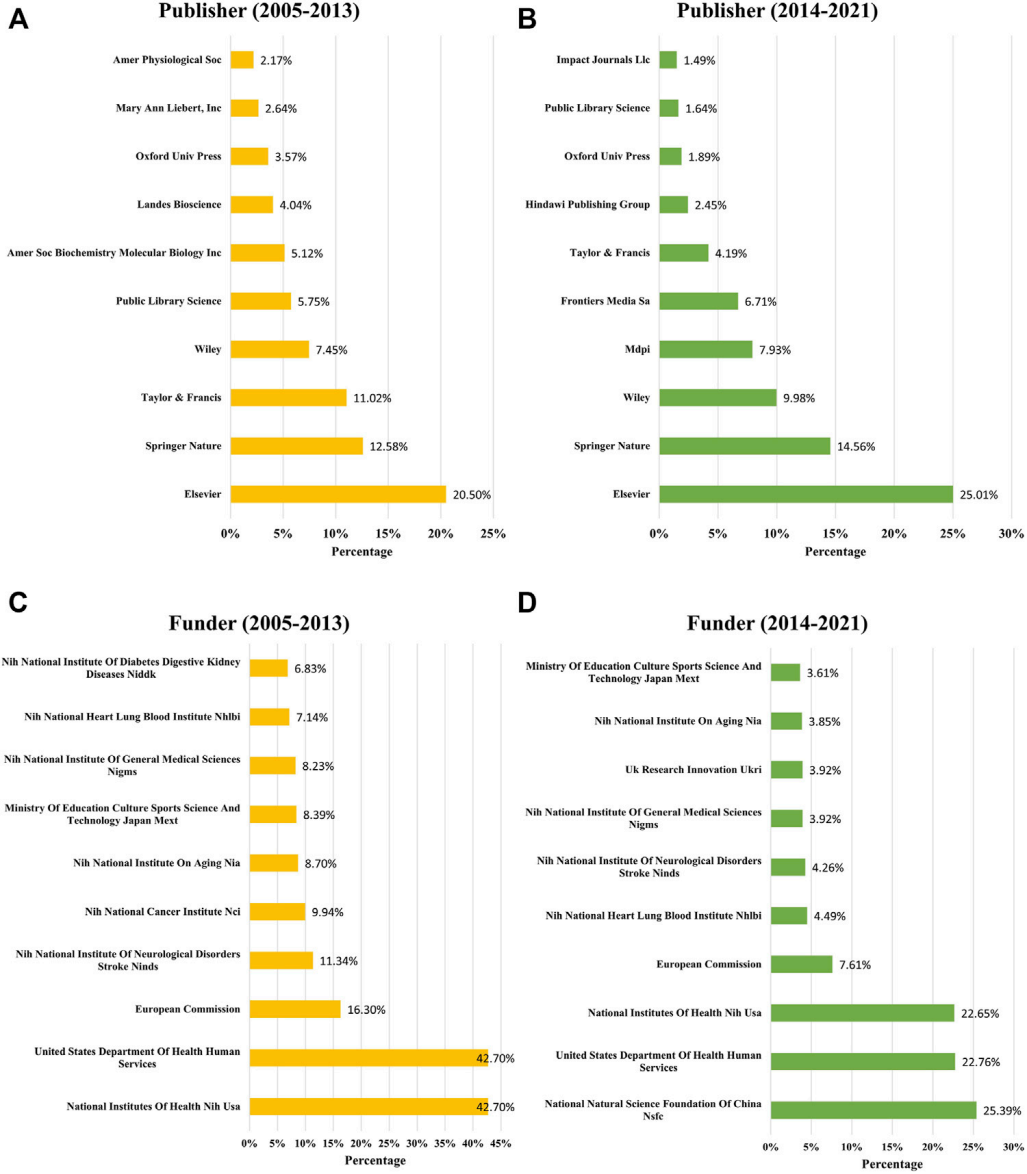
Publishers and funders changes for mitophagy research. Top 10 publishers with the largest number of articles on mitophagy during 2005–2013 **(A)** and 2014–2021 **(B)**. Top 10 funders sponsored the largest number of publications during 2005–2013 **(C)** and 2014–2021 **(D)**.

According to the published time, we divided the 5844 papers into two groups. There were 644 papers that were published during 2005–2013; and 5200 papers that were published during 2014–2021. Then the publishers and funders of each group were analyzed separately to show the trends over time. The top 10 publishers and funders of each group were presented in Figure 6. During 2005–2013, Elsevier (20.50%), Springer Nature (12.58%), Taylor and Francis (11.02%), Wiley (7.45%), and Public Library Science (5.75%) were the top five largest publishers in the field of mitophagy (Figure 6A). During 2014–2021, there was a huge increase in the number of publications on Mdpi (7.93%) and Frontiers Media Sa (6.71%), which ranked fourth and fifth, respectively (Figure 6B). It is noteworthy that Elsevier (25.01%) and Springer Nature (14.56%) were always the top two publishers in the two time periods.

During 2005–2013, over 40% of papers were funded by the National Institutes Of Health, NIH, United States (42.7%) or United States Department Of Health and Human Services (42.7%), all belong to the United States (Figure 6C). Other top five funders that sponsored the largest number of publications were the European Commission (16.30%), (NIH) National Institute Of Neurological Disorders and Stroke, NINDS (11.34%), and (NIH) National Cancer Institute, NCI (9.94%). While during 2014–2021, the National Natural Science Foundation Of China, NSFC, has become one of the most important funders and sponsored the largest number of publications (25.39%) (Figure 6D). Followed by the United States Department Of Health and Human Services (22.76%), National Institutes Of Health, NIH, United States (22.65%), European Commission (7.61%), and (NIH) National Heart, Lung, and Blood Institute NHLBI (7.61%).

### Top Cited Publications and Co-Cited References

The top-cited publications and co-cited references were fundamental and the basis of a certain field. In this study, the 10 most cited publications on mitophagy are listed in Table 4. All the top 10 publications were cited more than 900 times. The article (entitled A role for mitochondria in NLRP3 inflammasome activation) published in 2011 by Rongbin Zhou *et al.* obtained the largest number of citations (2,876). Of those, seven are articles (70%) and the rest are reviews (30%). As we can see from Table 4, all the top 10 most cited publications were published in high-impact journals before 2015. There were six papers with main themes on the functional roles of PINK1/Parkin and its related pathways in mitophagy. We suggested further reading these articles, if you wanted to get an in-depth knowledge of this field.

**TABLE 4:** Top 10 mitophagy-related publications with the most citations (up to 13 December 2021).

Rank	Title	Type	First author	Journal	Year	Citation	Major themes
1	A role for mitochondria in NLRP3 inflammasome activation	Article	Rongbin Zhou	Nature	2011	2,876	Reported a central role for mitophagy in the process of NLRP3 inflammasomes activation highlighting that mitochondria are essential for inflammatory response
2	Mechanisms of mitophagy	Review	Richard J Youle	Nature Reviews Molecular Cell Biology	2011	1,904	Comprehensively discussed the identified pathways that mediate mitophagy in yeast and mammalian cells and the role of mitophagy in Parkinson’s disease
3	PINK1/Parkin-mediated mitophagy is dependent on VDAC1 and p62/SQSTM1	Article	Sven Geisler	Nature Cell Biology	2010	1,763	Reported the functional role of PINK1/Parkin-mediated mitophagy through VDAC1 ubiquitination in the development of Parkinson’s disease
4	PINK1 Is Selectively Stabilized on Impaired Mitochondria to Activate Parkin	Article	Derek P Narendra	Plos Biology	2010	1,727	Provide a novel explanation for how PINK1 and Parkin work together to protect against damaged mitochondria by promoting mitophagy
5	Phosphorylation of ULK1 (hATG1) by AMP-Activated Protein Kinase Connects Energy Sensing to Mitophagy	Article	Daniel F Egan	Science	2011	1,606	Uncovers a mechanism that AMPK directly regulates mitophagy through phosphorylating and activating ULK1 thus establishing a direct molecular link between nutrient status and cell survival
6	The ubiquitin kinase PINK1 recruits autophagy receptors to induce mitophagy	Article	Michael Lazarou	Nature	2015	1216	Shows that PINK1 induces mitophagy directly, through the phospho-ubiquitin-mediated recruitment of NDP52 and OPTN, and that these receptors have an early role in recruiting the autophagy machinery
7	PINK1 stabilized by mitochondrial depolarization recruits Parkin to damaged mitochondria and activates latent Parkin for mitophagy	Article	Noriyuki Matsuda	Journal of Cell Biology	2010	1149	Describe the mechanism underlying the functional interplay between ubiquitination catalyzed by Parkin and mitochondrial quality control regulated by PINK1
8	PINK1-dependent recruitment of Parkin to mitochondria in mitophagy	Article	Cristofol Vives-Bauza	PNAS	2010	1068	Demonstrate that Parkin, together with PINK1, modulates mitochondrial trafficking, especially to the perinuclear region, a subcellular area associated with autophagy
9	The Roles of PINK1, Parkin, and Mitochondrial Fidelity in Parkinson’s Disease	Review	Alicia M Pickrell	Neuron	2015	1042	Summarize the functions of PINK1 and Parkin in normal cells, their molecular mechanisms of action, and the pathophysiological consequences of their loss
10	Autophagy, mitochondria and oxidative stress: cross-talk and redox signaling	Review	Jisun Lee	Biochemical Journal	2012	948	Summarize the basic mechanisms of mitophagy and the crosstalk between autophagy, redox signaling, and mitochondrial dysfunction highlighting its impact on chronic pathologies, particularly on neurodegenerative diseases

PNAS, Proceedings of the national academy of sciences of the United States of America.

**FIGURE 7:**
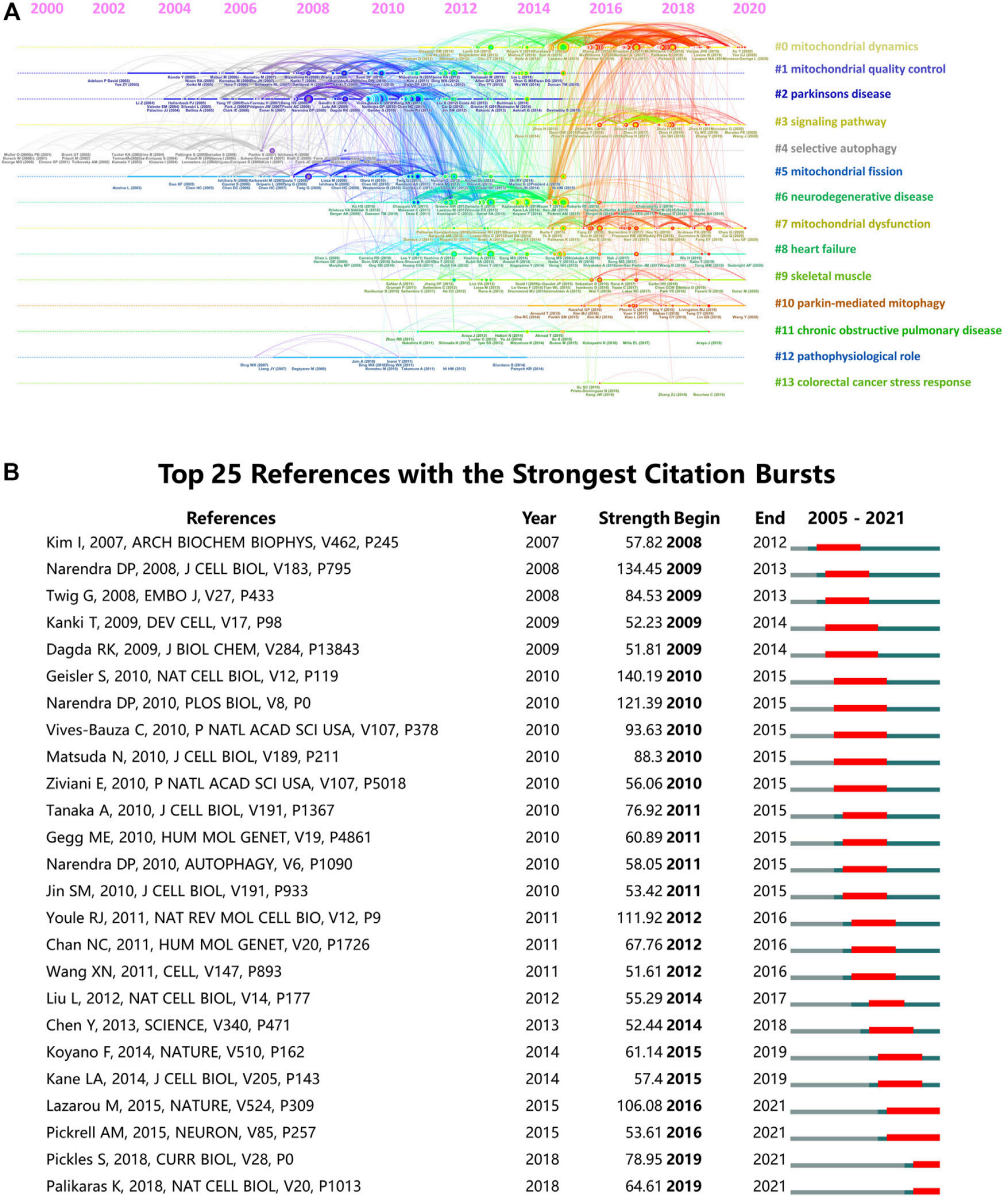
Analysis of co-cited references of the 5844 articles on mitophagy. **(A)** Timeline view of co-cited references. **(B)** Top 25 references with strongest citation bursts.

Next, we constructed a cluster analysis for all the co-cited references by CiteSpace software and presented the clusters in form of a timeline view in Figure 7A. Total 14 clusters were formed: #0 mitochondrial dynamics; #1 mitochondrial quality control; #2 Parkinson’s disease; #3 signaling pathway; #4 selective autophagy; #5 mitochondrial fission; #6 neurodegenerative disease; #7 mitochondrial dysfunction; #8 heart failure; #9 skeletal muscle; #10 parkin-mediated mitophagy; #11 chronic obstructive pulmonary disease; #12 pathophysiological role; #13 colorectal cancer stress response. Among them, “parkin-mediated mitophagy” was the most recent cluster. In addition, the top 25 references with the strongest citation bursts were identified by Citespace analysis (Figure 7B). Citation bursts represent a rapid rise in citations within a certain period, which can help characterize the dynamics of a research field (Amjad et al., 2022). In this study, the first burst of reference began in 2008. Among these 25 references, four references (28%) had a burst duration until 2021. The reference with the strongest burstiness (strength = 140.19) was published in *Nature Cell Biology* by Geisler S et al., in 2010.

### Analysis of Keywords

**FIGURE 8:**
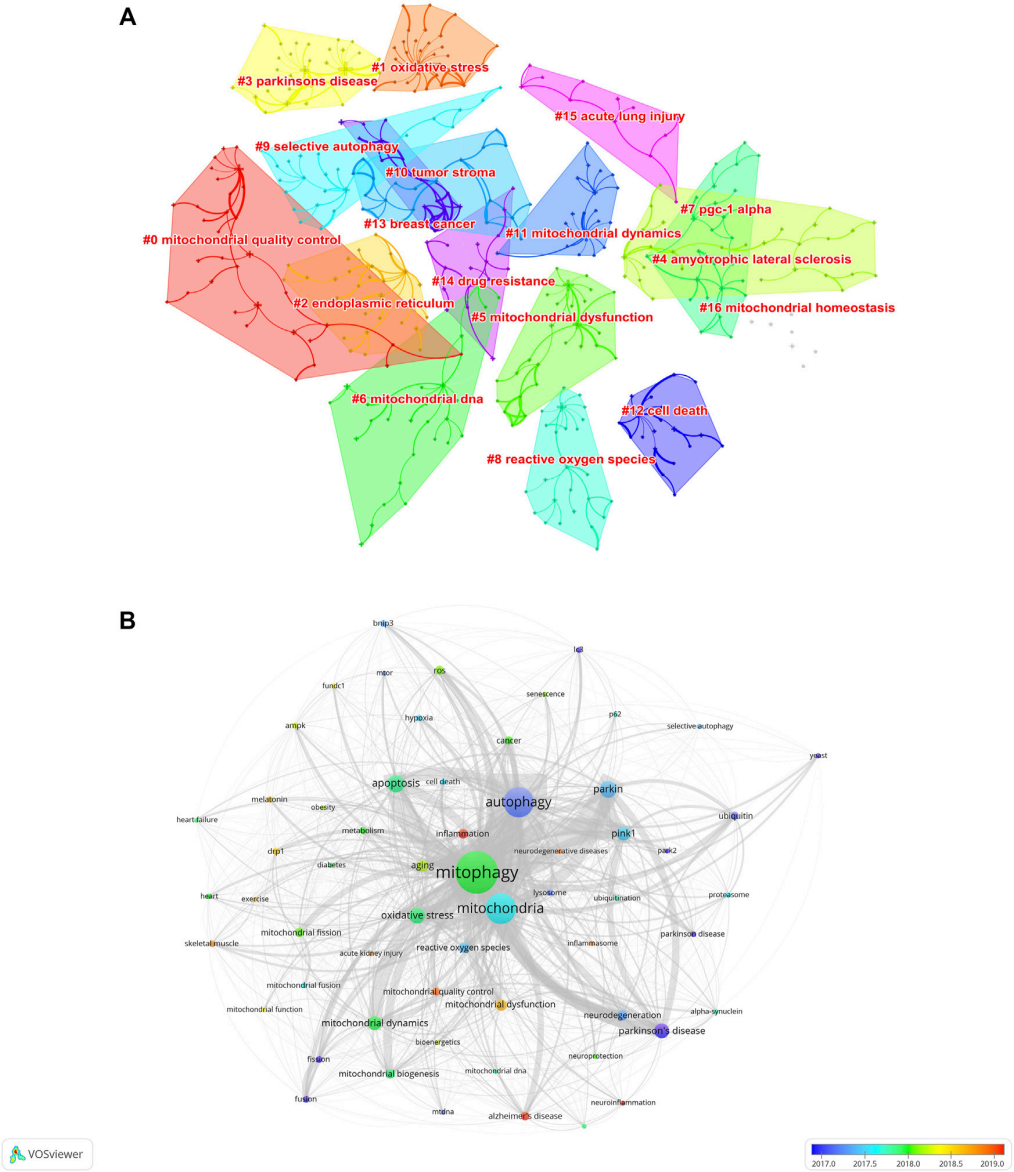
Analysis of keywords of the 5844 articles on mitophagy. **(A)** Clustering map of author keywords related to the research of mitophagy. **(B)** Author keyword overlay visualization map. The size of each circle indicates the frequency of occurrences of the author keyword. According to the color label in the lower right corner, the color of each circle indicates the average year when the keyword appeared in articles. The distance between any two circles is indicative of their co-occurrence link, and the thickness of the connecting line indicates the strength of the link.

To investigate the research hotspots in the field of mitophagy, a total of 7506 keywords (author keywords) were extracted from the 5844 publications for co-occurrence analysis on VOSviewer and Citespace software. Table 5 shows the top 30 high-frequency keywords. Among these keywords, “mitophagy” (2333 times) was the most frequently appeared one, followed by “mitochondria” (1242 times), “autophagy” (1185 timesand), “apoptosis” (410 times), and “parkin” (391 times). Keyword cluster analysis is based on the coexistence network to simplify the keywords in a small number of clusters. Through Citespace analysis, seventeen clusters were generated and listed as fellow: #0 mitochondrial quality control; #1 oxidative stress; #2 endoplasmic reticulum; #3 Parkinson’s disease; #4 amyotrophic lateral sclerosis; #5 mitochondrial dysfunction; #6 mitochondrial DNA; #7 PGC-1 alpha; #8 reactive oxygen species; #9 selective autophagy; #10 tumor stroma; #11 mitochondrial dynamics; #12 cell death; #13 breast cancer; #14 drug resistance; #15 acute lung injury; #16 mitochondrial homeostasis (Figure 8A and Table 6). Herein, the serial numbers are sorted by cluster size, and the clusters indicated that publications are grouped into different topics according to the keywords.

**TABLE 5:** Top 30 keywords related to mitophagy.

Rank	Keyword	Occurrences	Rank	Keyword	Occurrences	Rank	Keyword	Occurrences
1	Mitophagy	2333	11	Mitochondrial dysfunction	191	21	ubiquitin	101
2	Mitochondria	1242	12	Neurodegeneration	164	22	metabolism	83
3	Autophagy	1185	13	Reactive oxygen species	159	23	drp1	77
4	Apoptosis	410	14	Mitochondrial biogenesis	140	24	fission	73
5	Parkin	391	15	Inflammation	136	25	skeletal muscle	70
6	Oxidative stress	352	16	ROS	124	26	fusion	68
7	Parkinson’s disease	302	17	Mitochondrial fission	120	27	hypoxia	68
8	Pink1	275	18	Alzheimer’s disease	110	28	bnip3	66
9	Mitochondrial dynamics	267	19	Cancer	101	29	cell death	65
10	Aging	192	20	Mitochondrial quality control	101	30	ampk	61

**TABLE 6:** Top 17 largest clusters of keywords in the field of mitophagy.

Cluster ID	Size	Silhouette	Mean (Year)	Label	Cluster ID	Size	Silhouette	Mean (Year)	Label
0	33	0.99	2015	Mitochondrial quality control	9	24	0.99	2016	Selective autophagy
1	30	0.98	2014	Oxidative stress	10	21	0.97	2011	Tumor stroma
2	30	0.98	2016	Endoplasmic reticulum	11	21	1.00	2015	Mitochondrial dynamics
3	29	0.94	2014	Parkinson’s disease	12	19	0.99	2014	Cell death
4	29	0.94	2016	Amyotrophic lateral sclerosis	13	17	0.99	2012	Breast cancer
5	28	0.93	2013	Mitochondrial dysfunction	14	16	0.89	2015	Drug resistance
6	27	0.98	2016	Mitochondrial DNA	15	12	0.95	2016	Acute lung injury
7	26	0.94	2016	PGC-1 alpha	16	8	0.98	2018	Mitochondrial homeostasis
8	25	1.00	2016	Reactive oxygen species					

To further visualize the research frontiers in the field of mitophagy, the keyword co-occurrence analysis was performed on VOSviewer. In the overlay visualization map (Figure 8B), different colors of author keywords were marked according to the average publication years. For example, the keywords of “yeast’, “ubiquitin”, “park2”, “fusion”, “fission”, “autophagy”, and “Parkinson’s disease/Parkinson disease” were marked in blue, indicating that they were mainly found in the early years. While keywords such as “inflammation,” “Alzheimer’s disease,” “neuroinflammation”, “mitochondrial quality control”, “neurodegenerative disease”, “acute kidney injury”, skeletal muscle”, “exercise”, “drp1”, and “melatonin” marked in yellow or red, indicating that these keywords were represented the recent major topics in the field of mitophagy and may also become the frontiers in the future.

## Discussion

Nowadays, more and more research are being done around the world, and more and more scientific discoveries are being made. The biggest challenge is how to keep up with the remarkable explosion of knowledge in some active fields such as mitophagy. In this study, we took advantage of bibliometric analysis to visualize the global research landscape of mitophagy from 2005 to 2021. A total of 5844 articles were identified. Mitophagy research has flourished and attracted attention from all over the world, especially in United States, China, and the European Union. We found a list of outstanding institutions, researchers, journals, publishers, and funders which made a significant contributions to it. Research trends and hotspots were summarized and frontiers were predicted. Our findings provide a historical prospect and new insights into mitophagy.

### Trend in Countries/Regions

Although the annual number of publications on mitophagy research increased year by year, a regional imbalance in the development of mitophagy research was observed in this study (Figure 2B). With no doubt, United States, China, and the European Union were on the dominant position in the field of mitophagy (Figure 2B, Figure 4A, and Table 1). This could be partially explained by a large number of researchers and institutions and substantial fundings in them (Table 1 and Figure 6C). Previous studies suggested that economic progress and interregional cooperation are two important factors for promoting the development of research in a certain field (Wagner et al., 2018; Mormina, 2019). According to the GDP (https://datacatalog.worldbank.org/search/dataset/0038130), our data showed that high-income countries were more productive than low-income countries. This was consistent with other bibliometric studies conducted on mitophagy (Chen et al., 2021) and other topics, such as ferroptosis in cancer (Li et al., 2022), ATAC-seq (Zhao et al., 2020), intestinal microbiota in obesity (Yao et al., 2018), COVID-19 (Tantengco, 2021), and Viral Hepatitis (Ornos and Tantengco, 2022). Of note, although China and United States have a roughly equal number of publications, the degree of centrality was much lower in China (0.21) than that in United States (0.51). This indicated that United States put more emphasis on international cooperation, while China was more likely to conduct studies through national collaborative research.

### Knowledge Base and Landmarks

The knowledge base is the collection of co-cited references within the corresponding research community and the most cited articles are usually regarded as the landmarks (Chen et al., 2012; Zhang et al., 2021). In this bibliometric analysis of mitophagy, the top-cited articles and co-cited references were identified and reviewed as follows.

In 2005, the term mitophagy was firstly coined (Lemasters, 2005; Priault et al., 2005) for selective autophagy of mitochondria. In the early years, studies in yeast led to the discovery of several key proteins involved in mitophagy, such as Uth1p (Kissová et al., 2004), Aup1p (Tal et al., 2007), Mdm38 (Nowikovsky et al., 2007) and Atg32 (Kanki et al., 2009; Okamoto et al., 2009). In 2008, Derek demonstrated the role of Parkin protein in mediating the engulfment of mitochondria by autophagosomes and their subsequent degradation in mammalian cells (Narendra et al., 2008). And then, in 2010, four outstanding studies revealed the molecule mechanism of PINK1/Parkin-mediated mitophagy and its pathogenic mechanisms in the pathogenesis of Parkinson’s disease (Geisler et al., 2010; Matsuda et al., 2010; Narendra et al., 2010; Vives-Bauza et al., 2010). Hence, the functional links between PINK1 and Parkin as a classical mitophagy pathway were convincingly demonstrated. From then on, the role of mitophagy was massively studied and mitochondria were considered a promising therapeutical target for Parkinson’s disease (Malpartida et al., 2021). In that year, Ivana also identified the mitochondrial protein Nix (also known as BNIP3L) as a selective autophagy receptor in mammalian cells (Novak et al., 2010) and Atsushi Tanaka *et al* established the essential role of p97 in Parkin-mediated ubiquitination of mitofusins during mitophagy (Novak et al., 2010). These breakthroughs led to the mechanisms of mitophagy being initially established (Youle and Narendra, 2011). After that, other mechanisms such as AMPK-mediated phosphorylation of ULK1 (Egan et al., 2011), hypoxia-induced dephosphorylation of FUNDC1 (Liu et al., 2012), PINK1-dependent phosphorylation of ubiquitin (Koyano et al., 2014; Lazarou et al., 2015) were successively uncovered. In addition to the historic discovery of the functional links between mitophagy/autophagy and NLRP3 inflammasome activation (Zhou et al., 2011), these findings offer new insights into the mechanisms of mitophagy (Lee et al., 2012; Pickrell and Youle, 2015; Palikaras et al., 2018). In parallel, broader roles of mitophagy in other different diseases have attracted more and more attention after 2010 (Figure 7A).

### Hotspots Evolution and Frontiers

By analyses the keywords, research hotspots and trends can be effectively obtained (Wang H. et al., 2021; Zhang et al., 2021). The research trends on mitophagy can be roughly divided into three stages. During the first phase (2005–2010), researchers have focused mostly on the discoveries of new mechanisms in yeast and the roles of mitochondrial fission and fusion in neurodegenerative diseases (Priault et al., 2005; Kissova et al., 2006; Abeliovich, 2007; Tatsuta and Langer, 2008; Chen and Chan, 2009; Munakata and Klionsky, 2010). During the second phase (2011–2015), researchers’ attention have mostly been focused on the mitochondrial stress and dynamic network and roles of mitophagy in cancer, heart failure and related diseases (Egan et al., 2011; Dang, 2012; Dutta et al., 2013; Drew et al., 2014; Chourasia et al., 2015). During the third phase (2016–2021), researchers mostly attached attention to parkin-mediated mitophagy and its roles in skeletal muscle and inflammation-related diseases (Akabane et al., 2016; Kimura et al., 2017; Kravic et al., 2018; Lin et al., 2019; Han et al., 2020; Tran and Reddy, 2021). In all, we summarized the main research hotspots below: “mechanism of mitochondrial quality control”, “molecule and signaling pathway in mitophagy” and “mitophagy-related diseases”.

### Most Prolific and Influential Scientists

Top productive authors and top co-cited authors are the leading scientists in their respective fields. In this study, we found three scholars who were not only the top 10 productive authors but also the top 10 co-cited authors, namely Richard J. Youle, Hao Zhou, and Daniel J. Klionsky (Table 1). This implied their notable contributions to the field of mitophagy. Why can they be both prolific and influential? By further reading their publications, we can understand that Richard J. Youle mainly focused on the mechanisms of mitophagy (Youle and Narendra, 2011) and the roles of PINK1, Parkin, and mitochondrial fidelity in Parkinson’s Disease (Pickrell and Youle, 2015); Hao Zhou mainly focused on the role of mitophagy in cardiovascular disease, especially in cardiac microvascular ischemia/reperfusion injury (Zhou et al., 2018; Wang et al., 2020); Daniel J. Klionsky mainly focused on the selective mechanism during mitophagy (Kanki et al., 2009; Wang and Klionsky, 2011; Gatica et al., 2018). Their research directions were well matched with the research hotspots and frontiers of mitophagy. This may be one of the reasons why they can be the most important players in the research field of mitophagy.

## Conclusion

The present study was hardly not without limitations. First, this study queried only the WoSCC database. High-quality articles published in journals that are not included in the WoSCC database may be missed. The combination of Web of Science with other databases, such as PubMed, Google scholar, Dimensions, and Scopus, would enable a more robust bibliometric analysis. Second, this study also comes with certain limitations inherent in CiteSpace and VOSviewer software. For instance, some keywords in the articles may be not included in the analysis due to the incomplete keyword extraction methods and the clustering analysis based on only the main information (not the full text). Third, although we have standardized some terms that have different expression types, bias may still exist if other synonyms were available.

In summary, by bibliometric analysis of 5844 publications over the previous 17 years, we found that mitophagy research has flourished and attracted attention from all over the world, especially in the United States (2025, 34.65%) and China (1968, 33.68%). A list of outstanding institutions and scholars made a significant contribution, such as Chinese Acad Sci (112, 1.92%), Univ Pittsburgh (109, 1.87%), Richard J. Youle (N = 38), and Derek P. Narendra (co-citation: 2693). Many journals played an important role in promoting the development of mitophagy research, such as *Autophagy* (N = 208) and *Journal of Biological Chemistry* (co-citation: 17226). We summarized current research hotspots (“mechanism of mitochondrial quality control”, “molecule and signaling pathway in mitophagy”, and “mitophagy related diseases”) and predicted future frontiers (“parkin-mediated mitophagy” and its roles in “skeletal muscle” and “inflammation-related diseases”). This information will provide researchers with references for future mitophagy studies.

## Data Availability

The original contributions presented in the study are included in the article/supplementary material, further inquiries can be directed to the corresponding author.
